# Supplementary material for: Achillea millefolium L. and Achillea biebersteinii Afan. Hydroglycolic Extracts–Bioactive Ingredients for Cosmetic Use
Source: Molecules. 2020 Jul 24;25(15):3368. doi: 10.3390/molecules25153368 (PMC7436264; doi:10.3390/molecules25153368)
Supplement: Supplementary file 1 [file molecules-25-03368-s001.pdf]

molecules

Supplementary Materials for the Article

Achillea millefolium L. and *Achillea biebersteinii* Afan. Hydroglycolic Extracts–Bioactive Ingredients for Cosmetic Use

Katarzyna Gaweł-Bęben [1,*], Marcelina Strzępek-Gomółka [1], Marcin Czop [2], Zuriyadda Sakipova [3], Kazimierz Głowniak [1] and Wirginia Kukula-Koch [4]

[1] Department of Cosmetology, University of Information Technology and Management in Rzeszów, Sucharskiego 2, 35-225 Rzeszów, Poland; mstrzepek@wsiz.rzeszow.pl (M.S.-G.); kglowniak@wsiz.rzeszow.pl (K.G.)
[2] Department of Clinical Genetics, Medical University of Lublin, Radziwiłłowska 11, 20-080 Lublin, Poland; marcin.czop@umLub.pl
[3] School of Pharmacy, Kazakh National Medical University named after S.D. Asfendiyarov (KazNMU), 88 Tole bi street, 050012 Almaty, Kazakhstan; sakipova.z@kaznmu.kz
[4] Department of Pharmacognosy, Medical University of Lublin, Chodźki 1, 20-093 Lublin, Poland; virginia.kukula@gmail.com
* Correspondence: kagawel@wsiz.rzeszow.pl; Tel.: +48-17-866-1412

Academic Editor: Halina Ekiert and Agnieszka Szopa
Received: 30 June 2020; Accepted: 21 July 2020; Published: date

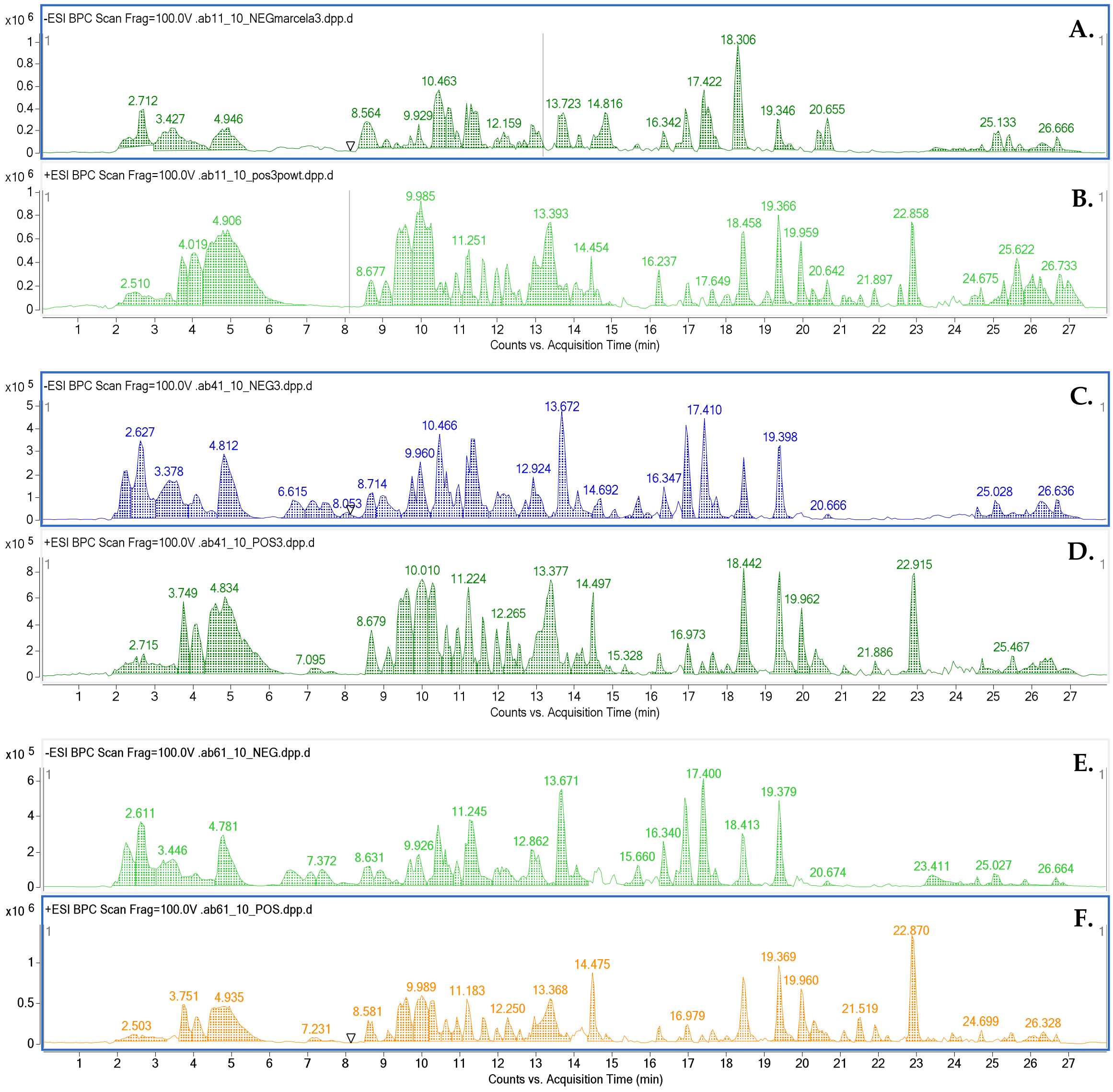

Figure S1. The TIC chromatogram recorded in the negative (A, C, E) and positive (B, D, F) ionization modes for the *A. biebersteinii* hydro-glycolic extract: HG 1:1 (A, B), HG 4:1 (C, D) and HG 6:1 (E, F).

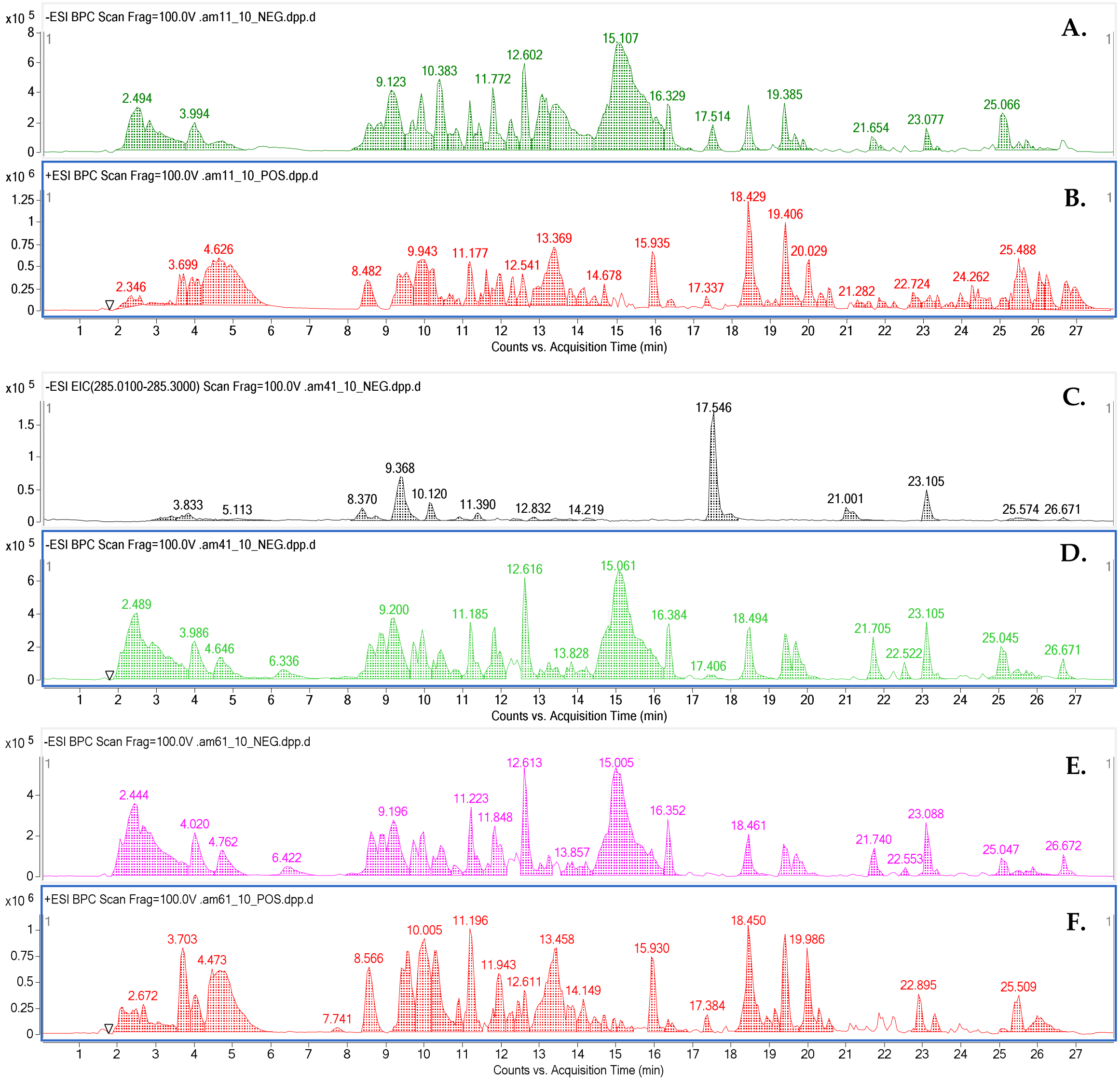

Figure S2. The TIC chromatogram recorded in the negative (A, C, E) and positive (B, D, F) ionization modes for the *A. millefolium* hydro-glycolic extract: HG 1:1 (A, B), HG 4:1 (C, D) and HG 6:1 (E, F).

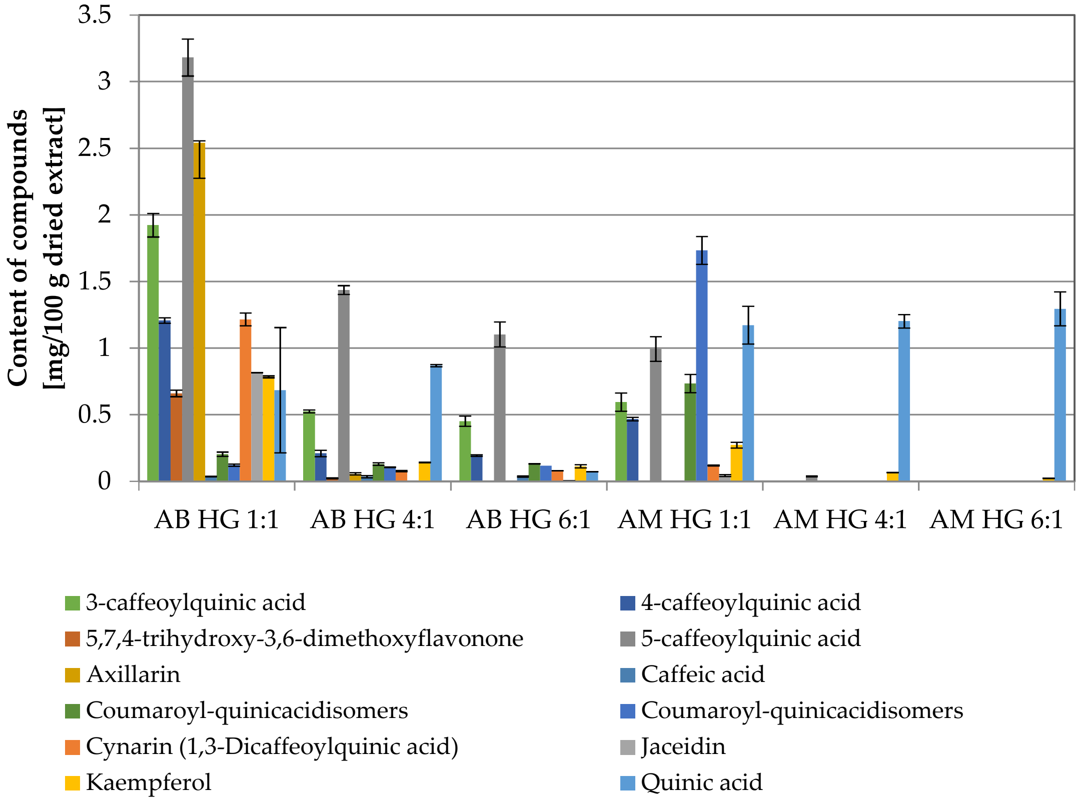

Figure S3. The quantitative composition of the studied extracts; values of graph represent mean ±SD; AM—*Achillea millefolium*, AB—*Achillea biebersteinii,* HG—hydroglycolic extract
